# The impact of Internet use on the subjective well-being of Chinese residents: From a multi-dimensional perspective

**DOI:** 10.3389/fpsyg.2022.950287

**Published:** 2022-08-11

**Authors:** Jiawei Zhong, Wenbo Wu, Fusen Zhao

**Affiliations:** ^1^Jing Hengyi School of Education, Hangzhou Normal University, Hangzhou, China; ^2^College of Business, Shanghai University of Finance and Economics, Shanghai, China

**Keywords:** Internet use, SWB, Chinese residents, multi-dimensional, heterogeneity

## Abstract

As cyberspace has become an important factor in modern-day life, the impact of the Internet on residents has also attracted more attention. Based on the data of China Family Panel Studies (CFPS), this study empirically examines the impact of Internet use on Chinese residents’ subjective well-being (SWB) from a multi-dimensional perspective. The research found that Internet use had a significant impact on residents’ SWB, which was mainly reflected in job satisfaction, happiness, social ties, and future confidence. The impacts of the Internet’s different application fields are not consistent. Applying its use more in studying, working, socialize and commercial activities has a stronger effect, but has no significant impact on entertainment. Further heterogeneity tests also found that the marginal effect of Internet use increases with age, male and low-income groups can obtain greater benefit from the Internet, and there is almost no difference between urban and rural areas. This research provides micro evidence of the social effects of Internet use and provides enlightenment for how to further promote the quality of Internet use to better benefit people.

## Introduction

The pursuit of subjective well-being (SWB) is the goal of economic development and people’s lives. Residents’ SWB not only reflects the satisfaction of individuals with their material and spiritual life, but also reflects the progress of a society. Since its reform and opening, China’s economy has continued to grow and people’s material living standards have been continuously improved. However, under the traditional development mode of pursuing economic growth, Chinese residents’ SWB has not increased significantly with the rapid economic growth. The World Happiness Report in 2022 gives the happiness index ranking of 146 countries and regions in the world. Among them, Finland has been rated as the happiest country in the world for five consecutive years by virtue of its proximity to nature, safety, and availability of services, while China ranks 72nd, only in the middle of the ranking. For this reason, in recent years, the Chinese government has attached great importance to the cultivation of residents’ SWB and regarded the construction of a happy society as one of the important tasks of China’s current economic and social development^1^. In the government work reports from 2016 to 2021, the Chinese government has mentioned “improving people’s SWB and making the people have more happiness and sense of gain” for many years^2^. At this stage, how to improve residents’ SWB has become an important topic in all sectors of society.

At the same time, with the advent of the era of the digital economy, a new generation of information technology represented by the Internet is being integrated into all walks of life, resulting in unprecedented changes to people’s productivity and lives. Thus, the following question arises: Does Internet use have a significant impact on residents’ SWB? In response to this issue, scholars in different fields have conducted extensive discussions (Morrell et al., 2004; Valkenburg et al., 2006; Brooks, 2015; Niu et al., 2018). On the one hand, Internet use helps to improve the happiness of residents. For example, the Internet enriches daily life, improves communication efficiency, creates consumption value, and increases income (Purcell et al., 2013; Campante et al., 2018). On the other hand, the use of the Internet may also have negative effects. For example, the Internet will reduce the scale of individuals’ offline social circle, increase depression and loneliness (Kraut et al., 1998), reduce offline emotional interaction, and reduce individuals’ enthusiasm to participate in social activities (Sabatini and Sarracino, 2017). Overall, there is no unified agreement as to whether Internet use is beneficial to the improvement of residents’ SWB. Most of the relevant studies have focused on developed countries, while there are relatively few studies of emerging countries. In recent years, the Internet has made great progress in China. According to the latest report released by the China Internet Network Information Center, by December 2021 the number of Internet users in China had reached 1.032 billion, and the Internet penetration rate had reached 73.0%. China has become the premier Internet country. At present, there are still few similar studies on whether Internet use has improved Chinese residents’ SWB, which undoubtedly restricts the effectiveness of constructing China’s happy society in the Internet age.

In view of this, this study uses the CFPS2018 study to explore the relationship between Internet use and residents’ SWB. Specifically, this study aims to examine the following questions: does Internet use affect residents’ SWB? Is the effect different due to individuals, family factors, and so on? Is there heterogeneity in the impact of different dimensions of Internet use (such as learning, work, entertainment, etc.) on residents’ SWB? Compared with the existing literature, the marginal contributions of this study are as follows. First, whether Internet use is beneficial, harmful, or indifferent for residents’ well-being is still inconclusive in developed countries. Based on Chinese practice, this study pays attention to the impact of Internet development on individuals in transition countries, and finds evidence that Internet use is conducive to improving Chinese residents’ well-being, which is an important supplement to the research field of the relationship between the Internet and individual SWB. Second, this study defines individual SWB from different dimensions, and comprehensively investigates its impact on individual SWB in combination with different fields of Internet use. Existing literatures mostly use single dimension indicators, such as life satisfaction or happiness, to represent SWB, and it is difficult to achieve a comprehensive investigation of the impact of the Internet on SWB. However, our research found that the effect of Internet use on SWB of different dimensions is not consistent, and at the same time, different purposes of Internet use also have a heterogeneous effect on SWB, which expands some static research results in developed countries. The findings are significant, and provides new empirical evidence for how Internet use affects residents’ SWB. Finally, against the background that informatization and human capital are increasingly important for future economic development (Heckman and Kautz, 2012; Yushkova, 2014; United Nations Conference on Trade and Development [UNCTAD], 2019), exploring the relationship between Internet use and individual SWB will not only help to deeply understand the evolution of individual subjective feelings in the information society, but also provide targeted enlightenment for the further deepening and popularization of Internet use.

The study is organized as follows. The first section reviews the progress of research into SWB. Then, Chinese and non-Chinese studies of the relationship between Internet use and residents’ SWB are reviewed. Next, the data, variables, and research methods are introduced, followed by the empirical results and discussions. Finally, the study provides the main research conclusions and enlightenment.

## Literature review

### Subjective well-being

Classical liberalism believes that SWB is an important indicator of economic measurement, which has the same utility as welfare. It is the great goal of all human efforts (Ng, 1996). In fact, the discussion of what constitutes SWB has a long history in China. Confucianism believes that “worry is joy and turn from worry into joy,” while Taoism adheres to “settle down in life outside worry and don’t turn worry into joy” (Cai, 1982; Chen, 2008). Until now, people have never reached a unified conclusion about the essence of SWB, but this does not affect the fact that SWB has become an eternal pursuit and a new topic of interest. In the 1950s, as society paid greater attention to residents’ welfare, psychologists and sociologists in Europe and America tried to study SWB for its disciplinary characteristics, and it quickly attracted the attention of a large number of scholars. In 1974, American economist Easterlin put forward the famous Easterlin paradox, and then increasing numbers of economists began to study SWB (Salanova et al., 2004; Purcell et al., 2013; Campante et al., 2018; Li and Zhou, 2021).

At present, the research on SWB has involved many fields, and different disciplines have different understandings. Psychologists study SWB from the perspective of behavioral science and have put forward three standards of the definition of SWB: “external standard,” “internal emotion,” and “individual self-evaluation” (Ryff, 1989). Among them, positive and negative emotions are important components of SWB, which are clearly different from each other but are significantly related (Diener et al., 2009). Positive emotions can improve individuals’ psychological function and provide effective protection for individuals to achieve a higher sense of SWB, while negative emotions have the opposite effect (Hicks et al., 2013). Correspondingly, sociologists have proposed “social well-being” and “psychological well-being” (Winkelmann, 2009). They are more concerned about the individual’s sense of satisfaction and recognition in social activities, that is, the social realization value, such as the social support and social trust (Rehdanz and Maddison, 2008; Prati et al., 2016). Economists’ research on SWB takes “rational people” as the premise, equates SWB with maximum utility, and usually uses more specific indicators such as life satisfaction and job satisfaction (Clark et al., 2008). Such studies often link SWB with economic indicators (Easterlin et al., 2010). For example, in a model for measuring SWB constructed by Branch (2004), the SWB index is set as the actual utility welfare, income level, emotion and Eigenfunctions associated with individuals.

In summary, SWB is a concept with multiple dimensions, and although there are different conceptual descriptions of SWB across disciplines, they are only different literally, empirical results using the self-report research method show that the internal structures of different concepts are consistent (Ryan and Deci, 2001; Nave et al., 2008). At the same time, there is a common core connotation in different concepts, that is, as a subjective experience, SWB is the overall emotional and cognitive evaluation and feeling of the quality of life made by individuals according to their standards, including their so-called happiness, life satisfaction, and so on (Diener, 1994; Ryff and Singer, 2008; Diener et al., 2017). In real life, individuals have various understandings of SWB, which is caused by various ideal aspirations, life attitudes, economic conditions and social environments. At this time, if individual’s SWB is only investigated from a single dimension, it is impossible to accurately judge and compare the possible heterogeneous effects of Internet use in different scenarios such as life, work, and self-evaluation. This study examines individual SWB from a multi-dimensional perspective, which can more comprehensively take into account the deviations caused by individual differences, so as to achieve a more scientific research and evaluation.

### The relationship between Internet use and subjective well-being

In the era of the digital economy, information technology represented by the Internet not only affects the operation of the whole national economy, but also affects all aspects of residents’ lives. Many researchers have begun to study the impact of the Internet on economic growth and residents’ lives. At the macro level, the Internet can eliminate market friction (Choi and Yi, 2009), promote the transformation of traditional enterprises (Pisano et al., 2015), and promote business innovation (Paunov and Rollo, 2016) and financial development (Xie et al., 2016). At the micro level, the Internet can change household or individual consumption decisions (Song and Zahedi, 2005), employment patterns (Feldman and Klaas, 2002), and timing (Tokunaga and Rains, 2010).

At present, the research on the relationship between Internet use and residents’ SWB has been widely involved in the fields of psychology, sociology, and economics, but no consistent conclusion has been reached. One research view holds that there is a “network gain effect,” that is, people can promote social participation and social capital accumulation through the Internet, and then improve their SWB (Morrell et al., 2004). Many studies have found that Internet use increases positive effects such as social communication, creating consumption value, and increasing income, thus verifying the network gain effect (Hong, 2007; Sabatini and Sarracino, 2017). Moreover, from a multi-dimensional perspective, Internet use has improved people’s positive feelings and life satisfaction (Sabatini, 2011; Martin and Omrani, 2015; Ganju et al., 2016; Lu and Kandilov, 2021). In terms of social networking, Internet platforms such as Facebook, QQ, and WeChat have played an important role in expanding interpersonal relationships, strengthening the accumulation of personal social capital, and finally improving users’ SWB (Steinfield et al., 2008; Graham and Nikolova, 2013; Niu et al., 2018). In terms of production and work, Internet use can promote workplace change at any time, such as a move to a home office or entrepreneurship, improve the processing efficiency of work data and daily life information, promote the improvement of the frequency of communication between individuals and colleagues, and finally improve individual SWB (Cilesiz, 2009; Castellacci and Vinas-Bardolet, 2019). In terms of daily life, on the basis of enriching people’s lifestyles, such as shopping, leisure, and entertainment, the popularity of the Internet has also improved the degree of information asymmetry, promoted the improvement of price discovery mechanisms, and reduced transaction costs, thus reducing commodity prices and significantly improving the welfare level of consumers (Hong, 2007; Long and Yi, 2019).

Another view is that Internet use reduces people’s face-to-face interpersonal interactions and social communication, thus reducing people’s trust value, triggering negative emotions, such as loneliness and fear, and then reducing SWB. This view is called the “substitution effect of presence” (Pénard et al., 2013; Hage et al., 2016). Brooks (2015) used the workplace as an example to verify existence of the “substitution effect of presence.” On the one hand, telecommuting may reduce the face-to-face interaction between colleagues or leaders, resulting in poor information communication. On the other hand, the use of social media at work can disrupt work order and reduce productivity, which can lead to lower job satisfaction. Antonucci et al. (2007) investigated the negative impact of Internet on SWB from life field. They believed that although Internet use provided online interaction opportunities for different individuals, it reduced the frequency and quality of face-to-face communication among family members, resulting in unfamiliar family relations, and disharmonious family relations often led to a reduction in quality of life and life satisfaction. Beyond that, based on the perspective of social comparison theory, the generation of individual SWB is often not only based on its own goal, but also compared with multiple standards to obtain information. When individuals compare themselves with those around them, if they feel that their working ability and income level are better than others, they will obtain higher SWB (Fujita and Diener, 1997; Park and Baek, 2018). According to Clark and Senik (2010), Internet use has expanded the reference group to which people can compare themselves. The reference object goes beyond the boundary of daily life and can be easily compared with others online from any country and any background. This comparison reduces the individual’s subjective positioning of their relative income, more seriously, the comparison behavior based on this will bring a sense of psychological loss and relative deprivation, which will undoubtedly lower people’s judgment of life satisfaction.

In the existing literature, the relationship between Internet use and SWB has been discussed more internationally, and the research perspectives are quite diverse, which provides an important reference for our research (Kraut et al., 1998; Valkenburg et al., 2006; Steinfield et al., 2008; Martin and Omrani, 2015; Castellacci and Vinas-Bardolet, 2019). In contrast, such literature using China as the sample still lags. Although a few studies have discussed the relationship between Internet use and residents’ SWB (Zhu and Leng, 2018; Long and Yi, 2019; Zhou and Zhang, 2021), the following problems still exist. First, the relevant literature has mostly used happiness to measure individual SWB. Few studies have comprehensively investigated the impact of Internet use on different dimensions of residents’ SWB (including job satisfaction, life satisfaction, happiness, social ties, and future confidence) under an analytical framework. Second, the method of measuring Internet use is relatively simple, and few studies have comprehensively investigated the possible heterogeneous impact of different areas of Internet use on residents’ SWB. Based on the above research gap, this study attempts to analyze the relationship between Internet use and residents’ SWB from a multi-dimensional perspective, and explores the heterogeneous impact caused by individual, family, and urban and rural factors in order to provide a supplement to the existing research.

## Data and method

### Data

The data used in this study was mainly obtained from the CFPS2018 study. The data was collected by the Institute of Social Science Survey Center of Peking University. The sample covered 25 provinces/cities/autonomous regions in China. The target sample size was 16,000 households. The survey objects included all family members in the sample households. It is nationally representative, micro-individual data in China. This study mainly focused on the impact of adult (individuals aged 16 and above) Internet use on individual SWB. Among them, Internet use, SWB, and demographic characteristic variables were taken from the CFPS2018 personal database, and a few other control variables were derived from the CFPS2018 family economic database. In the process of sample data processing, the samples with incomplete key data and abnormal values were eliminated, and finally 10,943 effective individual samples were obtained for benchmark testing.

### Estimation methods

In order to investigate the impact of Internet use on residents’ SWB, this study set the following benchmark empirical model:


(1) $$SWB_{i}=C_{i}+\text{α}Internet_{i}+\text{β}Control_{i}+u+{\text{ε}}_{i}$$

In the model, *SWB* is the explanatory variable; *Internet* is the core explanatory variable, which indicates the degree of individual use of the Internet; α is the influence coefficient of the core explanatory variable; *Control* represents a series of control variables that may affect residents’ SWB, including individual characteristics, family characteristics, and other factors; *i* represents individual residents; *u* represents the fixed effect at the county level, and ε is a random perturbation term.

### Variable description

#### Subjective well-being measures

Subjective well-being is a subjective feeling of people involving multiple dimensions. The existing research has not formed a unified standard for measurement of SWB. Based on the existing research (Brockmann et al., 2009; Hicks et al., 2013; Li and Zhou, 2021), this study measured individual SWB from five dimensions: job satisfaction, life satisfaction, happiness, social ties, and future confidence. This multi-dimensional comprehensive index is suitable for different categories of people and is more universal. According to the CFPS2018, these five dimensions correspond to five questions: “how satisfied are you with your work?,” “how satisfied are you with your life?,” “how happy are you?,” “how good are your relationships?” and “how confident are you in your future?” The answers to each question from “very low” to “very high” were scored on a scale of 1 to 5 points, respectively. Considering that individual SWB reflects a comprehensive and coordinated feeling, it was more appropriate to investigate it from an all-round perspective. Therefore, the research mainly used the equal weight method to construct a comprehensive SWB index.

These multi-dimensional indicators formed by these questions have sufficient theoretical basis for measuring SWB, and the method is generally applicable internationally. Theoretical evidence shows that the definition of SWB is becoming more and more diverse, and a single dimension of satisfaction cannot fully explain SWB, but should also include social relationships, self-esteem, and emotions (Morrell et al., 2004; Diener et al., 2009; Easterlin et al., 2010; Brooks, 2015; Niu et al., 2018). Ryff and Singer (2008) described SWB from six dimensions, including purpose of life, personal growth, positive relations with others, environmental mastery, autonomy, and self-acceptance. Recent review literatures, Çikrıkci (2016), Castellacci and Tveito (2018) summarized that SWB consists of different elements^3^. These views coincide with our understanding of SWB. Methodologically, judging from the existing literature, the databases on residents’ SWB in different countries can provide strong evidence for our research^4^. The measurement method of SWB in the national-level database we used is commonly used internationally (Alesina et al., 2004; Dolan et al., 2008; Pénard et al., 2013). The above facts can provide important evidence for our research. In this study, these dimensions we selected almost include the factors that are generally concerned in existing research, and can reasonably represent the Chinese residents’ SWB. Not only that, the Cronbach alpha value of our SWB scale is 0.726, indicating that it has sufficient reliability in terms of internal consistency.

#### Internet use

The main explanatory variable of this study is Internet use. In CFPS2018, the measurement of Internet use mainly includes five levels, corresponding to five questions, asking frequency of using Internet to studying, working, socialize, entertainment, and commercial activities. The answer options include “almost every day,” “3–4 times a week,” “1–2 times a week,” “2–3 times a month,” “once a month,” “once every few months,” and “never.” The different options were assigned a score on a scale of 1 to 7, respectively. Here, a comprehensive index able to reflect the degree of Internet use was obtained through the following steps. First, considering that the original assignment method presents a negative correlation between the higher the score and the lower the intensity of Internet use, in order to facilitate understanding, the scores were inverted so that the scores showed a positive correlation with the degree of Internet use. Second, through the correlation analysis of the indicators at five levels, it was found that the correlation coefficients of different indicators were mostly below 0.3, indicating that the collinearity relationship between different indicators is low. Finally, based on the degree of Internet use reflected by the five questions, each has its own focus, and the collinearity relationship between them is low. In order to better reflect the comprehensiveness of Internet use, the equal weight method was also used to construct comprehensive Internet use.

#### Control variables

In order to scientifically evaluate the impact of Internet use on SWB, this study selects the following variables for analysis based on existing research (e.g., Brooks, 2015; Castellacci and Vinas-Bardolet, 2019; Zhou and Zhang, 2021): individual age, gender, education level, health status, marital status, household registration, work experience, religious belief, and family income. Age is one of the important factors affecting SWB. Existing studies generally believe that due to work pressure, life expectations and other reasons, SWB may first decline and then rise with age (Ferrer-I-Carbonell and Gowdy, 2007). Gender and marital status are common influencing factors in happiness research literature. On average, men’s SWB is significantly lower than that of women (Alesina et al., 2004); Studies in different regions have found that the SWB of married residents is higher (Dolan et al., 2008). The level of education also has a direct or indirect impact on the individual’s SWB. The reason is that education level is an important factor affecting individual development. Education provides individuals with more opportunities to improve their income and social status, but it may also make people more reflective and thus generate more psychological stress, so the effect of education on SWB is uncertain (Clark and Oswald, 1994; Oshio and Kobayashi, 2010). It needs to be briefly explained here that in the evaluation of education, the scoring system was not completely continuous. A score from 3 to 9 indicated that the highest educational background of an individual was from primary school to doctoral level, 0 referred to illiterate/semi-illiterate, and 10 was never attended school. For convenience, the scores were adjusted from 0 to 5 to represent never attended school, primary school, junior middle school, high school, college, and undergraduate and graduate students, respectively. Health can significantly affect individual SWB. Generally, the better the physical health, the higher the SWB (Haller and Hadler, 2006). In addition, because the evaluation of health status also adopted the reverse scoring mechanism, that is, the higher the individual’s health level, the lower the score, the individual health scores were positively standardized in order to facilitate understanding. Work experience will have an important impact on people’s psychological endurance, and will also increase the breadth of individual social relations, thus affecting individual subjective feelings (Antonucci et al., 2007). Household registration in China has special characteristics, generally, people who live in urban area often means that they can enjoy more social and public services, and their SWB may also be higher (Long and Yi, 2019). Religious belief is also one of the important factors affecting SWB. Many researchers believe that religious belief can help individuals build confidence to face reality and provide moral constraints for individual behavior, thus affecting SWB (Dolan et al., 2008). Most of the literature on happiness economics confirms that income is one of the important factors affecting SWB (Blanchflower and Oswald, 2004; Boyce et al., 2010), so it is necessary to include it as one control variable.

The descriptive statistical results of all variables are shown in Table 1.

**TABLE 1:** Variable description and descriptive statistics.

Variable name	Variable meaning	Mean value	Standard error	Minimum value	Maximum value
*SWB*	Residents’ SWB	3.880	0.659	1	5
*Internet*	Degree of Internet use	0.358	0.193	0.143	1
*lnage*	Natural log of residents’ age	3.651	0.380	2.773	4.625
*gender*	Man = 1, woman = 0	0.547	0.498	0	1
*marriage*	Married = 1, unmarried = 0	0.616	0.486	0	1
*education*	Educational background	1.503	1.577	0	5
*health*	Health condition	0.420	0.237	0	1
*residence*	Rural residents = 0, urban residents = 1	0.236	0.425	0	1
*work*	Currently employed = 1, otherwise = 0	0.665	0.472	0	1
*religion*	Religious = 1, otherwise = 0	0.288	0.453	0	1
*lnincome*	Natural log of family per capita hourly income	1.109	0.642	0	4.772

## Estimation results and discussion

### Benchmark results’ analysis

When using econometric models to estimate unknown parameters in linear regression models, the ordinary least square (OLS) method is the most common and basic estimation method in regression models (Angrist and Pischke, 2008). It minimizes the sum of squares of the differences between the real dependent variables and the predicted dependent variables to obtain the best linear unbiased estimator. Because the OLS estimation results are intuitive and easy to interpret, many literatures on SWB directly use the OLS method (Brockmann et al., 2009; Knight et al., 2009; Zhou and Zhang, 2021). This study also used the OLS method to estimate the relationship between Internet use and residents’ SWB by gradually adding control variables. At the same time, considering the data characteristics of the explained variables, we also use the order probit model for estimation. It can be seen from the results that the influence of Internet use on SWB is significantly positive, but the *R*^2^ of OLS model is higher, which means that the OLS model is more suitable for this study, so we mainly use the results of OLS model for interpretation. The results are shown in Table 2. The results in column (1) do not include other control variables, and then in column (2) the individual and family variables are included. It can be seen that the coefficients of Internet use are positive and significant at the level of 1%. This means that Internet use has a positive impact on residents’ SWB. Column (3) controls the county-level fixed effect based on the inclusion of all control variables. The result is still significantly positive, indicating that the above results have good robustness. Overall, although previous studies on the effect of internet use on SWB have produced inconclusive results, the China case suggests that Internet use has a positive impact on residents’ SWB. In addition, after adding control variables and county-level fixed effects, the *R*^2^ of the model was greatly improved, which indicates that it is necessary to add control variables. According to the results of column (3) including all control variables, the coefficient of Internet use on residents’ SWB is 0.276. Hence, providing other conditions remain unchanged, an increase of 10% of Internet use can promote residents’ SWB by about 2.76%. From the results of the control variables, individual characteristics and family factors also affect residents’ SWB in different degrees, which is also consistent with the existing relevant research. There is a significant inverted U-shaped relationship between age and residents’ SWB. Health condition, marital status, urban–rural differences, employed or not, and family income all have an important impact on residents’ SWB.

**TABLE 2:** Basic results of Internet use on SWB.

	OLS			Oprobit		
	(1)	(2)	(3)	(4)	(5)	(6)
*Internet*	0.221***	0.258***	0.276***	0.352***	0.470***	0.529***
	(8.07)	(8.14)	(8.06)	(7.22)	(7.87)	(8.09)
*lnage*		−3.062***	−3.198***		−5.522***	−6.068***
		(−6.74)	(−6.79)		(−6.57)	(−6.90)
*lnage_sq*		0.443***	0.462***		0.802***	0.879***
		(6.88)	(6.93)		(6.72)	(7.05)
*gender*		−0.016	−0.011		−0.026	−0.017
		(−1.49)	(−0.96)		(−1.33)	(−0.82)
*marriage*		0.177***	0.174***		0.319***	0.329***
		(10.75)	(9.90)		(10.71)	(10.25)
*education*		−0.001	−0.001		−0.006	−0.005
		(−0.21)	(−0.15)		(−0.57)	(−0.47)
*health*		0.761***	0.725***		1.422***	1.418***
		(28.58)	(26.09)		(27.90)	(26.50)
*work*		0.008	0.023*		0.012	0.029
		(0.65)	(1.66)		(0.48)	(1.12)
*residence*		0.025**	0.028*		0.042*	0.050*
		(1.98)	(1.88)		(1.82)	(1.79)
*religion*		0.027**	0.029**		0.053***	0.059***
		(2.43)	(2.48)		(2.60)	(2.71)
*lnincome*		0.018**	0.034***		0.027*	0.058***
		(2.00)	(3.03)		(1.68)	(2.77)
*_cons*	3.770***	8.490***	8.727***			
	(315.24)	(10.74)	(10.57)			
*county_FE*	*No*	*No*	*Yes*	*No*	*No*	*Yes*
*N*	10943	10943	10943	10943	10943	10943
*R* ^2^	0.005	0.097	0.175	0.001	0.023	0.043

***, **, and *, respectively, mean significant at 1%, 5%, and 10% levels, and *t* values in bracket is clustered.

### Robustness test

#### Endogeneity

Endogeneity is the main problem to be solved in empirical analysis. This study examined the relationship between Internet use and residents’ SWB, and there will inevitably be possible endogenous problems. On the one hand, unobservable factors, such as regional culture, circumstances, or other factors, will affect individual Internet use and SWB at the same time. As these factors are unobservable, it was difficult to fully include them in the estimation model. This possible omission of variables could lead to bias in the estimation results. On the other hand, the data used in this study was the cross-sectional data of a single year, and individual SWB is the subjective feeling observed during the survey, which may change with Internet use, resulting in reverse causality problems. Therefore, it was essential to solve the potential endogenous problems. The study used the following two methods to solve this problem.

(1) Instrumental variable method. Based on the existing literature, this study took the country-level Internet information infrastructure and the average degree of Internet use as the instrumental variables of individual Internet use. The reason for this was that, on the one hand, the usage of information technologies such as the Internet often have a peer effect. According to the way the Internet transmits information, the residents’ Internet use is often closely related to the local information infrastructure, in which computers and mobile phones are the most common hardware tools. On the other hand, as a macro level factor, the computer or mobile phone utilization rate at the county level is hardly affected by the Internet usage frequency at the micro individual level. Therefore, this study constructed two instrumental variables from the county level. First, the average level of computer utilization in the county, which was expressed by the ratio of the total number of respondents using computers to the total number of respondents in the county. Second, the average level of Internet use in the county, expressed by the average level of Internet use of all respondents in the county where the individual is located. Table 3 reports the results. It can be seen that there is an obvious positive correlation between the instrumental variables and Internet use, which means that they meet the correlation hypothesis, and the coefficient of Internet use is still significantly positive. This result is consistent with the result of benchmark testing.

**TABLE 3:** Robustness test results.

	Instrumental variable method	Introduce lagging Internet use	PSM method		Replace explanatory variable
	(1)	(2)	(3)	(4)	(5)
*Internet*	0.259***		0.059***	0.056***	
	(8.23)		(3.23)	(3.16)	
*Internet*_2016		0.152***			
		(3.22)			
*Internet*1					0.066***
					(11.59)
*lnage*	−3.092***	−3.703***	−3.024***	−3.024***	−0.014
	(−5.12)	(−5.38)	(−3.25)	(−3.25)	(−1.31)
*lnage_sq*	0.463***	0.532***	0.453**	0.453**	0.170***
	(5.10)	(5.43)	(2.09)	(2.09)	(9.70)
*gender*	−0.015	−0.003	−0.139***	−0.139***	0.004
	(−1.07)	(−0.21)	(−2.68)	(−2.68)	(0.70)
*marriage*	0.159***	0.156***	−0.116	−0.116	0.720***
	(6.96)	(7.08)	(−1.52)	(−1.52)	(26.03)
*education*	−0.002	0.010	0.533***	0.533***	0.019
	(−0.10)	(1.36)	(11.43)	(11.43)	(1.35)
*health*	0.709***	0.720***	−0.060	−0.060	0.029*
	(10.72)	(19.45)	(−0.46)	(−0.46)	(1.96)
*work*	0.025	0.032*	0.579***	0.579***	0.028**
	(1.10)	(1.77)	(8.25)	(8.25)	(2.38)
*residence*	0.044	0.031*	0.515***	0.515***	0.035***
	(1.36)	(1.70)	(7.82)	(7.82)	(3.15)
*religion*	0.027**	0.027*	0.050	0.050	8.962***
	(2.44)	(1.84)	(1.34)	(1.34)	(10.80)
*lnincome*	0.032**	0.037***	0.083***	0.083***	−0.014
	(2.00)	(2.63)	(7.90)	(7.90)	(−1.31)
*_cons*	3.720***	9.623***	−1.404***	−1.404***	0.170***
	(10.56)	(8.05)	(−2.78)	(−2.78)	(9.70)
*IV*1	0.609***				
	(25.76)				
*IV*2	0.068***				
	(8.48)				
First stage *F* value	473.75				
*county_FE*	*Yes*	*Yes*	*Yes*	*Yes*	*Yes*
*N*	10943	6771	10404	10404	10943
*R* ^2^	0.175	0.182	0.254	0.254	0.184

***, **, and *, respectively, mean significant at 1%, 5%, and 10% levels, and *t* values in bracket is clustered.

(2) Introduction of lagged Internet use. Considering the possible reverse causality between variables when using cross-sectional data analysis, this study replaced the key explanatory variables with the Internet use in CFPS2016 without changing the explanatory variables and control variables. Introducing lag explanatory variables can show that Internet use is earlier than SWB, and this can solve the possible reverse causality problem to a certain extent. According to the results in column (2) of Table 3, after the reverse causality problem was solved by introducing the lag explanatory variable, the coefficient of Internet use is still significantly positive, which verifies the benchmark test results again.

#### Other robustness tests

(1) Propensity Score Matching (PSM) method. This method can analyze the causal relationship between Internet use and SWB based on the observed data and can alleviate possible selective errors. The basic process was as follows. First, the conditional probability of individuals entering the experimental group (high-frequency Internet use group) was obtained through a Probit model, that is, the so-called propensity score. In this study, a series of control variables in the benchmark test model were selected as explanatory variables for the Probit model. Second, the experimental and control groups were matched according to the propensity score, and a balance test was performed. If the test is passed, there is only significant difference between the two groups in the key variables, and there is no difference in other control variables. Here, the minimum nearest neighbor matching method and radius matching method were used. Finally, observing the differences between different groups of samples obtains the average treatment effect. If the effect is still significant and positive, the above test results can be verified. The results of the balance test show that after propensity score matching, there is no systematic difference between different groups. The results in columns (3) and (4) in Table 3 show that the coefficients of Internet use obtained by different matching methods are consistent and significantly positive.

(2) Replace explanatory variable. In the previous calculation of Internet use, this study fully considered the situation of using the Internet in different fields and constructed a comprehensive index. In CFPS2018, there is also a single dimension index that can be used to indirectly measure the degree of individual Internet use. The corresponding question is “the importance of the Internet to your access to information,” and the corresponding answers are assigned scores of 1 to 5 from “very unimportant” to “very important,” respectively. Obviously, individuals believe that the importance of using the Internet to obtain information can reflect their degree of Internet use to a certain extent. Therefore, this study used this index to replace the comprehensive index of Internet use in the previous article. Combined with the results in column (5) of Table 3, after replacing the explanatory variables, Internet use still has a positive effect on residents’ SWB.

From the above results, it can be seen that after solving endogeneity problem and robustness tests, the influence coefficient of Internet use on SWB is still significantly positive, which indicates that Internet use significantly improves the SWB of Chinese residents. Such results validate the “network gain effect,” which corresponds to Hong (2007); Sabatini and Sarracino (2017)’s research, that is, Internet use has increased social communication, created consumer value, increased income and other positive effects. So far, this study has solved a basic problem, that is, whether the Internet has improved the SWB of Chinese residents, but we still need to solve a deeper problem, that is, from a multi-dimensional perspective, what aspects of the Internet will affect SWB? The previous literature review shows that the Internet will not only enhance people’s social circle, but also may be conducive to improving people’s work efficiency. At the same time, it will also have an important impact on daily life. Therefore, we need to do more analysis work.

### Multi-dimensional test

Through the above analysis and the variable construction process, the SWB constructed in this study included five dimensions, which, respectively, reflect the individual’s job satisfaction, life satisfaction, happiness, positive relationship with others, and confidence in the future. The question then arises: what dimensions of SWB are affected by Internet use? The comprehensive index of Internet use includes different aspects of learning, work, entertainment, social and business activities. Is there a significant difference in the impact of individuals’ Internet use in different fields on SWB? Next, this study further explored the impact of Internet use on residents’ SWB from the perspective of different dimensions of SWB and different dimensions of Internet use.

#### Different dimensions of subjective well-being

The comprehensive indicators used to measure SWB were sub-divided into five sub-indicators for testing. This multidimensional study responds the review points of Çikrıkci (2016), Castellacci and Tveito (2018), they believe that SWB consists of different elements. Firstly, SWB is assessed under the hedonic dimension of well-being. It’s an assessment of their own life by the individual in terms of obtaining satisfaction; secondly, psychological well-being develops on the basis of the eudaimonic dimension of SWB. It deals with the potential of the individual to enter interactions with others using abilities and communication skills and the responsibilities of all these processes in terms of life aims; thirdly, self-esteem is also the component of SWB, it comprises all internal beliefs of the individual about themselves. This study measured individual SWB from five dimensions: job satisfaction, life satisfaction, happiness, social ties, and future confidence. These views coincide with our understanding of SWB. According to the results of the impact on different dimensions of well-being in Table 4, there are certain differences in the impact of Internet use on different dimensions of SWB. Specifically, Internet use has significantly promoted residents’ job satisfaction, happiness, social ties, and future confidence, but has no significant impact on life satisfaction. The possible reason is that, on the one hand, the efficient matching mode brought by the Internet enables people to choose their own work according to their work preferences, and the workplace and working methods are more diversified. At the same time, the instant messaging function of the Internet solves communication problems caused by distance (Purcell et al., 2013), which makes individuals benefit from the Internet. On the other hand, while increasing online communication, Internet use also reduces face-to-face communication between family members and reduces the quality of face-to-face interaction, resulting in tension in family relations (Sabatini and Sarracino, 2017). Disharmonious family relations will lead to the decline of quality of life and life satisfaction. We also used Oprobit model for analysis, and the conclusion is consistent. See Appendix Table A1 for the test results.

**TABLE 4:** Results of different dimensions of SWB.

	*Job satisfaction*	*Life satisfaction*	*Happiness*	*Social ties*	*Future confidence*
	(1)	(2)	(3)	(4)	(5)
*Internet*	0.422***	0.013	0.409***	0.378***	0.292***
	(7.61)	(0.23)	(3.51)	(7.71)	(5.92)
*lnage*	−6.766***	−3.917***	−15.743***	−2.933***	0.825
	(−8.97)	(−5.03)	(−9.38)	(−4.30)	(1.11)
*lnage_sq*	0.979***	0.561***	2.148***	0.445***	−0.137
	(9.17)	(5.11)	(9.10)	(4.63)	(−1.30)
*gender*	−0.079***	−0.033*	0.067*	0.009	0.061***
	(−4.37)	(−1.84)	(1.75)	(0.59)	(3.56)
*marriage*	0.068**	0.353***	1.122***	0.096***	0.179***
	(2.42)	(11.65)	(17.27)	(3.85)	(6.51)
*education*	0.021**	−0.006	0.058***	0.015*	−0.034***
	(2.25)	(−0.66)	(2.86)	(1.78)	(−3.71)
*health*	0.662***	0.877***	1.877***	0.474***	0.887***
	(14.42)	(19.49)	(18.91)	(11.91)	(20.65)
*residence*	0.121***	−0.046**	0.059	0.007	−0.013
	(5.30)	(−2.04)	(1.23)	(0.37)	(−0.62)
*work*	0.087***	0.070***	0.097*	−0.014	−0.031
	(3.73)	(2.89)	(1.91)	(−0.67)	(−1.36)
*religion*	0.042**	−0.003	0.008	0.025	0.051***
	(2.22)	(−0.15)	(0.21)	(1.52)	(2.86)
*lnincome*	0.055***	0.034*	0.054	0.021	0.026
	(3.02)	(1.84)	(1.35)	(1.34)	(1.51)
*_cons*	14.561***	9.949***	34.182***	8.064***	2.335*
	(11.02)	(7.24)	(11.54)	(6.70)	(1.80)
*county_FE*	*Yes*	*Yes*	*Yes*	*Yes*	*Yes*
*N*	10943	10943	10943	10943	10943
*R* ^2^	0.132	0.149	0.176	0.104	0.139

***, **, and *, respectively, mean significant at 1%, 5%, and 10% levels, and *t* values in bracket is clustered.

#### Different perspectives of Internet use

Internet use was divided into five sub-areas, which represent Internet use for learning, work, social, entertainment, and business activities. For now, the Internet has profoundly changed society, and has pervaded every corner of people’s lives. According to previous researches, the influence effects brought by Internet are different or even opposite, which may be related to people’s application of Internet to different fields (Ganju et al., 2016; Lu and Kandilov, 2021). As mentioned above, on the one hand, Internet improves work efficiency, which may improve SWB of individuals to a certain extent (Purcell et al., 2013; Campante et al., 2018). On the other hand, Internet may make people deeply immersed in online social interaction, thereby reducing face-to-face communication with family members, which is not conducive to SWB (Sabatini and Sarracino, 2017). Therefore, when Internet is used in different fields, it may have different effects on SWB, at this time, it is necessary to analyze the heterogeneity. Table 5 reports the empirical results. It can be seen that there are also significant different effects of SWB brought about by the application of the Internet in different fields. Among them, the higher the frequency of using the Internet for studying, working, socialize, and commercial activities, the stronger the promotion effect on residents’ SWB, corresponding regression coefficients are 0.139, 0.123, 0.090 and 0.045, respectively, and the above coefficients have passed the significance test at the 5% level. Comparing the results in different fields, it can be seen that the use of Internet for learning has the strongest effect on improving SWB, followed by the use of Internet for work, and finally the use of Internet for business activities, while the frequent use of the Internet for entertainment did not have an obvious promotion effect. This demonstrates that there is also “quantity” and “quality” in the field of Internet use. Using the Internet for their own reasons and then obtaining knowledge to improve individual skills will help cultivate residents’ SWB. On the contrary, being controlled by the Internet and indulging in entertainment will not have a positive effect on individuals’ SWB. We also used Oprobit model for analysis, and the conclusion is consistent. See Appendix Table A2 for the test results.

**TABLE 5:** Results of different areas of Internet use.

	(1)	(2)	(3)	(4)	(5)
*Internet for studying*	0.139***				
	(7.85)				
*Internet for working*		0.123***			
		(7.45)			
*Internet for socialize*			0.090***		
			(4.70)		
*Internet for entertainment*				0.021	
				(1.22)	
*Internet for commerce*					0.045**
					(2.05)
*lnage*	−3.132***	−3.188***	−3.338***	−3.254***	−3.273***
	(−6.65)	(−6.76)	(−7.02)	(−6.87)	(−6.91)
*lnage_sq*	0.450***	0.458***	0.483***	0.468***	0.471***
	(6.74)	(6.87)	(7.17)	(6.99)	(7.02)
*gender*	−0.016	−0.010	−0.010	−0.012	−0.012
	(−1.45)	(−0.94)	(−0.91)	(−1.09)	(−1.08)
*marriage*	0.172***	0.173***	0.171***	0.170***	0.170***
	(9.78)	(9.84)	(9.70)	(9.64)	(9.64)
*education*	0.003	0.001	0.010*	0.011*	0.009
	(0.55)	(0.09)	(1.76)	(1.86)	(1.64)
*health*	0.724***	0.727***	0.725***	0.726***	0.725***
	(26.06)	(26.11)	(26.02)	(26.02)	(26.01)
*residence*	0.020	0.011	0.023*	0.026*	0.025*
	(1.45)	(0.81)	(1.68)	(1.85)	(1.81)
*work*	0.028*	0.026*	0.036**	0.038**	0.037**
	(1.89)	(1.75)	(2.39)	(2.57)	(2.51)
*religion*	0.028**	0.029**	0.030***	0.030**	0.029**
	(2.37)	(2.51)	(2.58)	(2.56)	(2.49)
*lnincome*	0.038***	0.036***	0.041***	0.045***	0.044***
	(3.43)	(3.17)	(3.68)	(3.99)	(3.94)
*_cons*	8.689***	8.782***	8.948***	8.883***	8.921***
	(10.51)	(10.62)	(10.75)	(10.69)	(10.75)
*county_FE*	*Yes*	*Yes*	*Yes*	*Yes*	*Yes*
*N*	10943	10943	10943	10943	10943
*R* ^2^	0.174	0.174	0.172	0.170	0.170

***, **, and *, respectively, mean significant at 1%, 5%, and 10% levels, and *t* values in bracket is clustered.

### Heterogeneity test

From the above analysis, it is apparent that Internet use has played an important role in promoting residents’ SWB. The question that needs to be given attention is: will there be some differences in this impact due to individual, family, and other factors? Next, this study investigated heterogeneity from three aspects: individual age, family income, and urban–rural differences. We use OLS and Oprobit model to analyze respectively, and the results are consistent. The Oprobit model’s results can be seen in Appendix Table A3.

#### Comparative analysis of different age groups

Age is an important factor affecting SWB (Ferrer-I-Carbonell and Gowdy, 2007). Are there differences in the impact of Internet use on SWB among different age groups? Generally, young people are accustomed to using Internet for communication, work and study, and they will use Internet more frequently. On the contrary, the Internet is a new thing for the elderly, and their cognition of Internet is still being explored. Considering the differences of different age groups’ understanding and user experience of the Internet, this study divided the groups according to the age of the residents. Individuals aged 30 and below were regarded as the youth group, individuals aged 30–45 were regarded as the youth and middle-aged group, and individuals aged 45 and above were categorized as the middle-aged and above group. According to the results in Table 6, Internet use can significantly promote the SWB of different groups. For the youth group, the influence coefficient of Internet use on SWB is 0.217. For the youth and middle-aged group, the influence coefficient is 0.314. For the middle-aged and above group, the influence coefficient is 0.384. Compared with the increase of age, the promotion of Internet use on SWB is also increasing, that is, Internet use has the strongest effect on SWB in the middle-aged and above group, and the weakest effect in the youth group. We may be able to find possible reasons for this with sample data, and it shows that the middle-aged group and below prefer to use Internet more for study and work, while the middle-aged and above group use the Internet more for business activities and study. It means youth group are a generation growing up with the Internet, while enjoying the convenience of Internet, they also bear more pressure on study and work. For the middle-aged and above, the Internet appeared in the latter half of their lives, so it is more special, and they can obtain a greater marginal effect. At the same time, we provide interaction effects of age with internet use in Appendix Table A4, it shows that interaction effect is still significantly positive, which can verify above results.

**TABLE 6:** Heterogeneity test results.

	Different age groups			Different income groups		Different regional groups		Different gender groups	
	Age 30 and below	Age 30–45	Age 45 and above	Low-income	High-income	Urban	Rural	Female	Male
	(1)	(2)	(3)	(4)	(5)	(6)	(7)	(8)	(9)
*Internet*	0.217***	0.314***	0.384***	0.367***	0.185***	0.232***	0.316***	0.198***	0.347***
	(3.68)	(5.99)	(4.30)	(7.20)	(3.80)	(4.40)	(6.73)	(3.78)	(7.26)
*lnage*	−1.219	−0.508	9.064	−2.713***	−3.548***	−3.588***	−3.131***	−1.848**	−3.947***
	(−0.43)	(−0.09)	(1.17)	(−4.55)	(−4.30)	(−3.64)	(−5.62)	(−2.13)	(−6.74)
*lnage_sq*	0.153	0.079	−1.036	0.396***	0.509***	0.507***	0.454***	0.276**	0.567***
	(0.35)	(0.11)	(−1.07)	(4.70)	(4.35)	(3.68)	(5.75)	(2.23)	(6.86)
*gender*	0.001	−0.002	−0.060**	−0.024	0.005	0.006	−0.020	0.000	0.000
	(0.07)	(−0.13)	(−2.42)	(−1.62)	(0.28)	(0.32)	(−1.43)	(.)	(.)
*marriage*	0.154***	0.175***	0.235***	0.137***	0.214***	0.197***	0.166***	0.161***	0.179***
	(6.08)	(5.16)	(4.68)	(5.56)	(8.15)	(6.38)	(7.57)	(5.55)	(7.61)
*education*	0.007	−0.024**	0.042**	−0.012	0.013	0.008	−0.008	−0.001	0.004
	(0.53)	(−2.37)	(2.14)	(−1.47)	(1.43)	(0.85)	(−0.98)	(−0.14)	(0.48)
*health*	0.742***	0.783***	0.652***	0.746***	0.662***	0.690***	0.727***	0.719***	0.740***
	(14.14)	(17.58)	(12.10)	(21.32)	(13.73)	(13.16)	(21.84)	(16.31)	(19.74)
*work*	0.037	−0.007	0.033	0.024	0.012	0.014	0.016	0.013	0.027
	(1.36)	(−0.33)	(1.21)	(1.46)	(0.44)	(0.44)	(1.03)	(0.59)	(1.41)
*residence*	0.048*	0.046*	−0.022	0.051**	−0.007	0.000	0.000	0.013	0.044**
	(1.82)	(1.88)	(−0.69)	(2.33)	(−0.33)	(.)	(.)	(0.54)	(2.13)
*religion*	0.037*	0.037**	0.003	0.049***	−0.001	−0.002	0.045***	0.026	0.035**
	(1.76)	(2.01)	(0.10)	(3.18)	(−0.07)	(−0.12)	(3.07)	(1.43)	(2.15)
*lnincome*	0.000	0.055***	0.041*	0.036	0.068***	0.045**	0.031**	0.043**	0.022
	(0.01)	(2.95)	(1.67)	(1.61)	(2.88)	(2.35)	(2.09)	(2.42)	(1.44)
*_cons*	5.731	3.886	−16.325	7.881***	9.276***	9.359***	8.602***	6.284***	10.018***
	(1.29)	(0.40)	(−1.06)	(7.51)	(6.43)	(5.37)	(8.84)	(4.14)	(9.72)
*county_FE*	*Yes*	*Yes*	*Yes*	*Yes*	*Yes*	*Yes*	*Yes*	*Yes*	*Yes*
*N*	3461	4611	2871	6773	4170	3338	7605	4674	6269
*R* ^2^	0.264	0.218	0.194	0.183	0.244	0.234	0.190	0.198	0.214

***, **, and *, respectively, mean significant at 1%, 5%, and 10% levels, and *t* values in bracket is clustered.

#### Comparative analysis of different family incomes

In this study, those whose family income was below the average value were regarded as low-income family groups, and those whose family income was above the average value were regarded as high-income groups. According to the results of columns (4) and (5) in Table 6, the coefficient of Internet use on the SWB of low-income families is 0.367, which is significant at the level of 1%. The coefficient of Internet use on the SWB of high-income families is 0.185, which is only significant at the level of 5%. This result shows that compared with high-income families, Internet use plays a more significant role in improving the well-being of low-income families. To some extent, this reflects the complementary effect of the Internet and the advantages of new generation technologies such as the Internet. Specifically, China is a society of human relations and resources. In the period in which the Internet was underdeveloped, wealthy families often had more social resources and opportunities to obtain better work and lives. The rapid development of the Internet provided more opportunities for fair competition. Moreover, it also had the function of social supervision, which enabled poor families to obtain more benefits.

#### Comparative analysis of urban and rural areas

Considering the inherent dual structure of urban and rural areas in China, Internet use may have different effects on the SWB of residents in different regions. Therefore, this study used urban residents’ samples and rural residents’ samples for comparative analysis. According to the test results of columns (6) and (7) in Table 6, the impact of Internet use on the SWB of urban residents is 0.232, and the coefficient of rural residents’ SWB is 0.316, both of which are significant at the level of 1%. This shows that Internet use has an important impact on residents’ SWB in different regions, and there is little difference. This is partly due to the popularity of mobile Internet in China. According to the above data, China’s Internet penetration rate had reached 73.0% in 2022. In recent years, the Chinese government has continuously promoted the construction of “Broadband Countryside,” “Digital Finance” and other projects in order to achieve common prosperity and achieve the overall goal of a moderately prosperous society. Consequently, there is no significant difference in the popularity of the Internet between towns and villages. Therefore, there is no substantive difference in the SWB of residents in different regions.

#### Comparative analysis of different gender groups

Gender is one of the important factors affecting SWB, and the basic test results also verify this view. Is there a difference in the impact of Internet on the SWB of different genders? To examine this issue, this study divides the research sample into female group and male group. According to the results of columns (8) and (9) in Table 6, the coefficient of Internet use on the SWB of the female group is 0.198, and the coefficient in male group is 0.347. This result shows that Internet use has a greater effect on the improvement of males’ SWB. Considering the possible reasons, in China, males tend to bear more pressure from work and family life with the traditional family and social values, and they need to spend most of their energy on studying, living and working, so their SWB is generally lower than that of women (Basic results can verify this fact), Internet use provides them with convenience in work, study and life, so they gain more marginal effects. In addition to this, we provide interaction effects of gender with internet use in Appendix Table A4, it shows that interaction effect is still significantly positive, which can verify above results.

## Conclusion

With the deep integration of information technology in many aspects of people’s work, life, and entertainment, the impact of Internet use on micro individuals is increasingly becoming an important issue of concern for all sectors of society. SWB is not only an eternal goal pursued by people, but also an important indicator of social progress. Using the data of CFPS2018, this study discussed the impact of Internet use on residents’ SWB, which provides an important basis at the micro level for evaluating the economic and social effects of the Internet. The conclusions of this study are as follows: (1) Internet use has played a significant role in promoting residents’ SWB. This conclusion is still valid after solving endogenous problems and a series of robustness tests. (2) The promotion effect of Internet use on residents’ SWB is mainly reflected in job satisfaction, happiness, social ties, and future confidence, but the effect on life satisfaction is not significant. Individuals use the Internet more in the fields of studying, working, and socialize activities, which has a significant effect on the improvement of residents’ SWB, while using the Internet more for entertainment has no significant impact. (3) The promoting effect of Internet use on residents’ SWB is heterogeneous due to individual and family factors, and there is no significant difference between urban and rural areas. Specifically, Internet use has a stronger promoting effect on the SWB of older, male groups and has a more obvious impact on the SWB of low-income families.

This study examined the micro effects of Internet use, not only by analyzing the relationship between Internet use and residents’ SWB from the perspective of different dimensions, but also by exploring the heterogeneity of Internet use affecting residents’ SWB. This provides beneficial enlightenment for further improving residents’ SWB and deepening Internet application.

The policy implications of this study are as follows. First, with the rapid development of the digital economy, when building a digital society and a network-based power, the government should fully consider the impact of the Internet on micro individuals, so as to grasp “hard power,” on the one hand, by expanding the construction of network infrastructure and “soft power,” on the other hand, by helping people master more Internet skills, so that more people can deeply enjoy achievements gained through Internet use. Second, individuals’ correct understanding of the Internet should be established, and they should be actively guided to use it rationally. Only scientific and effective Internet use will have a more positive effect on micro individuals. This is particularly relevant for youth groups, because they are the source of human capital in the future. They should be guided to use the Internet reasonably and scientifically for study, work, and other aspects of life that can improve their skills or knowledge, and they should try to avoid indulging in online games and too much virtual social networking, so as to lay a foundation for the improvement of the quality of human capital in the future. Finally, the applicability of the Internet in different fields and regions should be deepened and expanded. The online classroom should be promoted for students, expanding the diversification of subjects and types of online education, and encouraging them to participate widely in online learning. For working groups, digital labor platforms can be promoted to explore the potential of the Internet for improving work efficiency and flexibility. In addition, equality of Internet resources between urban and rural areas should be promoted.

## Data availability statement

Publicly available datasets were analyzed in this study. This data can be found here: http://www.isss.pku.edu.cn/cfps/.

## Author contributions

JZ contributed to the writing – original draft, methodology, visualization, and resources. FZ contributed to the writing – review and editing, methodology, and formal analysis. All authors contributed to the article and approved the submitted version.

## Footnotes

1 At the first session of the 12th National People’s Congress of China in 2013, President Xi emphasized the close relationship between people’s SWB and the Chinese dream; The report at the 19th National Congress of the Communist Party of China (CPC) in 2017 also indicated that we should pay more attention to the SWB of ordinary people and take the people’s yearning for a betterr life as the goal of the Communist Party of China. At the Fourth Plenary Session of the 19th Central Committee of the CPC in 2019, President Xi also closely linked the SWB with the goal of achieving common prosperity.

2
http://www.gov.cn/gongbao/2022/issue_9766.htm

3 Firstly, hedonistic dimension, it’s an assessment of their own life by the individual in terms of obtaining satisfaction; secondly, psychological dimension, it deals with the potential of the individual to enter interactions with others using abilities and communication skills and the responsibilities of all these processes in terms of life aims; thirdly, dimensions of self-esteem, it comprises all internal beliefs of the individual about themselves.

4 In United States General Social Survey (GSS), Euro-barometer Survey Series, European Value Survey (EVS), World Values Survey (WVS), UK’s Annual Population Survey (APS) and other national survey databases, it is not difficult to find that similar related problems, which are widely used to measure life satisfaction, job satisfaction, happiness, living conditions and social capital to represent SWB.

## Appendix

**APPENDIX TABLE A1:** Results of different dimensions of SWB (Oprobit model).

	*Job satisfaction*	*Life satisfaction*	*Happiness*	*Social ties*	*Future confidence*
	(1)	(2)	(3)	(4)	(5)
*Internet*	0.547***	−0.006	0.187***	0.552***	0.385***
	(7.70)	(−0.08)	(2.82)	(7.80)	(5.56)
*Control variables*	*Yes*	*Yes*	*Yes*	*Yes*	*Yes*
*county_FE*	*Yes*	*Yes*	*Yes*	*Yes*	*Yes*
*N*	10943	10943	10943	10943	10943
*R* ^2^	0.057	0.065	0.052	0.049	0.069

***, **, and *, respectively, mean significant at 1%, 5%, and 10% levels, and *t* values in bracket is clustered.

**APPENDIX TABLE A2:** Results of different areas of Internet use (Oprobit model).

	(1)	(2)	(3)	(4)	(5)
*Internet for studying*	0.270***				
	(8.06)				
*Internet for working*		0.240***			
		(7.71)			
*Internet for socialize*			0.168***		
			(4.71)		
*Internet for entertainment*				0.032	
				(1.00)	
*Internet for commerce*					0.081**
					(1.98)
*Control variables*	*Yes*	*Yes*	*Yes*	*Yes*	*Yes*
*county_FE*	*Yes*	*Yes*	*Yes*	*Yes*	*Yes*
*N*	10943	10943	10943	10943	10943
*R* ^2^	0.043	0.043	0.042	0.042	0.042

***, **, and *, respectively, mean significant at 1%, 5%, and 10% levels, and *t* values in bracket is clustered.

**APPENDIX TABLE A3:** Heterogeneity test results (Oprobit model).

	Different age groups			Different income groups		Different regional groups		Different gender groups	
	Age 30 and below	Age 30–45	Age 45 and above	Low-income	High-income	Urban	Rural	Female	Male
	(1)	(2)	(3)	(4)	(5)	(6)	(7)	(8)	(9)
*Internet*	0.476***	0.623***	0.683***	0.393***	0.185***	0.501***	0.597***	0.380***	0.683***
	(3.97)	(6.15)	(7.27)	(3.95)	(3.80)	(4.61)	(6.85)	(3.79)	(7.51)
*Control variables*	*Yes*	*Yes*	*Yes*	*Yes*	*Yes*	*Yes*	*Yes*	*Yes*	*Yes*
*county_FE*	*Yes*	*Yes*	*Yes*	*Yes*	*Yes*	*Yes*	*Yes*	*Yes*	*Yes*
*N*	3461	4611	2871	6773	4170	3338	7605	4674	6269
*R* ^2^	0.072	0.055	0.047	0.044	0.066	0.047	0.063	0.051	0.054

***, **, and *, respectively, mean significant at 1%, 5%, and 10% levels, and *t* values in bracket is clustered.

**APPENDIX TABLE A4:** The interaction effects of age, gender with Internet use.

	OLS		Oprobit	
	(1)	(2)	(3)	(4)
*Internet*	0.256	0.162***	0.454	0.294***
	(0.67)	(3.62)	(0.62)	(3.48)
*Internet_lnage*	0.181*		0.321**	
	(1.78)		(2.40)	
*Intennet_gender*		0.202***		0.416***
		(3.65)		(3.97)
*lnage*	−3.205***	−3.265***	−6.095***	−6.211***
	(−6.52)	(−6.92)	(−6.62)	(−7.04)
*lnage_sq*	0.463***	0.472***	0.882***	0.900***
	(6.77)	(7.06)	(6.89)	(7.20)
*gender*	−0.011	−0.083***	−0.017	−0.166***
	(−0.96)	(−3.44)	(−0.83)	(−3.68)
*marriage*	0.174***	0.173***	0.329***	0.328***
	(9.84)	(9.88)	(10.18)	(10.22)
*education*	−0.001	0.000	−0.005	−0.003
	(−0.14)	(0.01)	(−0.46)	(−0.29)
*health*	0.725***	0.728***	1.418***	1.426***
	(26.09)	(26.22)	(26.49)	(26.63)
*work*	0.017	0.018	0.029	0.031
	(1.24)	(1.32)	(1.11)	(1.19)
*residence*	0.028*	0.029*	0.050*	0.053*
	(1.87)	(1.96)	(1.77)	(1.88)
*religion*	0.029**	0.028**	0.059***	0.057***
	(2.48)	(2.40)	(2.70)	(2.61)
*lnincome*	0.034***	0.034***	0.058***	0.057***
	(3.03)	(2.98)	(2.76)	(2.70)
*_cons*	8.743***	8.880***		
	(9.90)	(10.72)		
*county_FE*	*Yes*	*Yes*	*Yes*	*Yes*
*N*	10943	10943	10943	10943
*R* ^2^	0.175	0.176	0.043	0.044

***, **, and *, respectively, mean significant at 1%, 5%, and 10% levels, and *t* values in bracket is clustered.
